# Detection of asymptomatic malaria in Asian countries: a meta-analysis of diagnostic accuracy

**DOI:** 10.1186/s12936-022-04082-0

**Published:** 2022-02-16

**Authors:** Cho Naing, Norah Htet Htet, Saint Nway Aye, Htar Htar Aung, Marcel Tanner, Maxine A. Whittaker

**Affiliations:** 1 International Medical University, Kuala Lumpur, Malaysia; 2 James Cook University, College of Public Health, Medical and Veterinary Sciences, Douglas, QLD Australia; 3 Swiss Tropical and Public Health Institute (Swiss TPH), Basel, Switzerland; 4 University of Basel, Basel, Switzerland

## Abstract

**Background:**

Achieving malaria elimination requires the targeting of the human reservoir of infection, including those patients with asymptomatic infection. The objective was to synthesise evidence on the accuracy of the rapid-onsite diagnostic tests (RDTs) and microscopy for the detection of asymptomatic malaria as part of the surveillance activities in Asian countries.

**Methods:**

This was a meta-analysis of diagnostic test accuracy. Relevant studies that evaluated the diagnostic performance of RDTs and microscopy for detection of asymptomatic malaria were searched in health-related electronic databases. The methodological quality of the studies included was assessed using the QUADAS-2 tool.

**Results:**

Ten studies assessing RDT and/or microscopy were identified. The diagnostic accuracies in all these studies were verified by PCR. Overall, the pooled sensitivities of RDT, as well as microscopy for detection of any malaria parasites in asymptomatic participants, were low, while their pooled specificities were almost ideal. For the detection of *Plasmodium falciparum,* pooled sensitivity by RDT (59%, 95%CI:16–91%) or microscopy (55%, 95%CI: 25–82%) were almost comparable. For detection of *Plasmodium vivax,* pooled sensitivity of RDT (51%, 95% CI:7–94%) had also the comparable accuracy of microscopy (54%, 95%CI,11–92%). Of note are the wide range of sensitivity and specificity.

**Conclusion:**

The findings of this meta-analysis suggest that RDTs and microscopy have limited sensitivity and are inappropriate for the detection of asymptomatic *Plasmodium* infections. Other methods including a combination of PCR-based strategies, Loop-Mediated Isothermal Amplification (LAMP) technique must be considered to target these infections, in order to achieve malaria elimination. However, more data is needed for the wide acceptance and feasibility of these approaches. Studies to explore the role of asymptomatic and sub-patent infections in the transmission of malaria are of critical importance and are recommended.

**Supplementary Information:**

The online version contains supplementary material available at 10.1186/s12936-022-04082-0.

## Background

Many malaria endemic countries are now entering the pre-/elimination phase of malaria control. The elimination phase aims to bring local transmission to zero indigenous cases of a specified malaria parasite [1, 2]. There are concerns about the uneven progress in those countries with a high malaria burden. Achieving malaria elimination requires the targeting of the human reservoir of infection, including those patients with asymptomatic infection [3]. As it is critically important to detect and treat all malaria infections early so that they do not generate secondary cases [4, 5], more targeted surveillance is required in the elimination phase [4–6]. As such, the system employed in the elimination phase must have sufficiently high sensitivity to detect individual infections within low prevalence areas. The World Health Organization (WHO) recommends the use of rapid antigen-detection diagnostic tests (RDTs) in places where microscopy is not available thereby enabling enhanced case-based surveillance [5]. While high-quality RDTs are recommended and have been used as the standard diagnostic tool for routine malaria case management and passive case detection (PCD), it has been revealed that the accuracy of case detection by RDT in low transmission settings was questionable [7, 8]. Therefore, the objective of the meta-analysis was to synthesize evidence on the accuracy of the currently and routinely used field-based diagnostic tests (microscopy, RDTs) for detection of asymptomatic malaria as part of the surveillance activities in countries entering the pre-/elimination phase.

## Methods

### Search strategy

Relevant studies were searched electronic databases of Medline, EMBASE, Web of Science, the Latin American and Caribbean Health Sciences Literature (LILACS) and African Journals Online (AJOL) for relevant studies published in English until May 2021. The search was conducted using keywords and Boolean operators: (“malaria” OR “plasmodium”) AND (“dipsticks” OR “RDT” OR “rapid diagnosis” OR “rapid onsite diagnosis” OR “ICT” OR “immunochromatographic”) OR (“microscopy” OR “PCR”). We also checked the references of retrieved articles and relevant reviews manually for any additional studies. A search strategy in PubMed, as an example was ((((("diagnosis"[MeSH Terms] OR "diagnosis"[All Fields] OR "diagnostic"[All Fields]) AND accuracy[All Fields] AND rapid[All Fields] AND ("research design"[MeSH Terms] OR ("research"[All Fields] AND "design"[All Fields]) OR "research design"[All Fields] OR "test"[All Fields])) OR ("microscopy"[MeSH Terms] OR "microscopy"[All Fields])) AND ("malaria"[MeSH Terms] OR "malaria"[All Fields])) AND asymptomatic[All Fields]) AND ("epidemiology"[Subheading] OR "epidemiology"[All Fields] OR "surveillance"[All Fields] OR "epidemiology"[MeSH Terms] OR "surveillance"[All Fields]).

### Study selection

Human studies based on the guideline for diagnostic test accuracy (DTA) review [9] were selected (Additional File 1).

#### Type of studies

Any study design was included, if it had evaluated RDT/microscopy in participants residing in Asian countries.

#### Participants

Asymptomatic cases living in the malaria-endemic countries, detected by active case detection (ACD), reactive case detection (RCD) or survey. Asymptomatic cases were as defined in the primary study.

#### Index test

Any type of RDTs or routine microscopy for diagnosis of malaria,

#### Comparator test

There was either no test or test with alternative RDT/microscopy.

#### Target conditions

Human asymptomatic malaria cases, regardless of parasite species.

#### Reference standard

PCR.

#### Outcomes

These were the sensitivity and the specificity of the diagnostic test of interest. Sensitivity refers to the probability that the index test result will be positive in an infected case. Specificity refers to the probability that the index test result will be negative in a non‐infected case [10].

### Exclusion criteria

Studies that that focussed on symptomatic cases, pregnant women or travellers were excluded. Moreover, studies without sufficient data had to be excluded as well.

### Study selection and data extraction

Two investigators (SNA, CN) individually checked the titles and abstracts, and then selected the relevant full-text articles, according to the inclusion criteria. The two investigators independently extracted information from each included study, using a pre-tested data extraction form prepared for this review. Information collected comprised author, year of publication, country, participant characteristics (e.g., mean age, male %), study design characteristics (e.g., design, settings, sample size, calendar period of study, study mode such as ACD, RCD, survey), diagnostic tests’ characteristics (e.g., test name, brand, samples used) and the test performance results (e.g., sensitivity, specificity). Disagreements between the two investigators were resolved by consensus.

### Assessment of methodological qualities

Two investigators (NHH, CN) independently assessed study quality following the Quality Assessment of Diagnostic Accuracy Studies-2 (QUADAS-2) tool [11], appropriately tailored to fit in this review. The four domains such as patient selection, index test, reference standard and flow and timing were applied. Each domain was assessed by using the ‘signalling questions’ (e.g., “was a case–control design avoided?”) with ‘yes’, ‘no’, or ‘unclear’ as an answer. The answers to these ‘signalling questions’ were then used to judge whether the risk of bias was low and if the study was applicable to the meta-analysis. If the response to the risk of bias and applicability questions were ‘low risk’ or ‘low concern’, the articles were given one point each.

### Statistical analysis

Study-specific test accuracy was estimated in terms of sensitivity and specificity along with their 95% confidence intervals (CIs). TP, TN, FP, and FN represent the number of true positives, true negatives, false positives and false negatives, respectively.

$$ \begin{gathered} {\text{sensitivity}} = {\text{ TP}}/ \, \left( {{\text{TP}} + {\text{FN}}} \right){\text{ and}} \hfill \\ {\text{specificity}} = {\text{ TN }}/ \, \left( {{\text{TN}} + {\text{FP}}} \right) \hfill \\ \end{gathered} $$

The pooled estimates of sensitivity and specificity were calculated using a random-effects bivariate model. These bivariate random-effects modelling of sensitivity and specificity produced the pair of performance measures that are interdependent [12–14]. Separate analyses were carried out for microscopy and RDT.

### Heterogeneity analysis

Heterogeneity was statistically assessed using the *I*^2^ test. The *I*^2^ test values describe the percentage of total variation across studies that is attributable to the heterogeneity rather than chance. *I*^2^ value greater than 50% is regarded as substantial heterogeneity [15].

### Subgroup analysis

Analysis was stratified by type of index test, either RDT or microscopy. It was planned to do subgroup analyses to assess the effect of age group, the methodological quality of studies, RDT brands and study country. However, due to the small number of studies or limited data in the studies, it was not possible to do so.

As there is no validated method for publication bias in test accuracy reviews available at present [16], this was not done. All statistical analyses were done with the ‘*Midas*’ programme in STATA 14 (STATACorp, Tx) and the QUADAS-2 tool was with RevMan 5.3 (The Nordic Cochrane Centre, Copenhagen, Denmark).

## Results

**Fig. 1:**
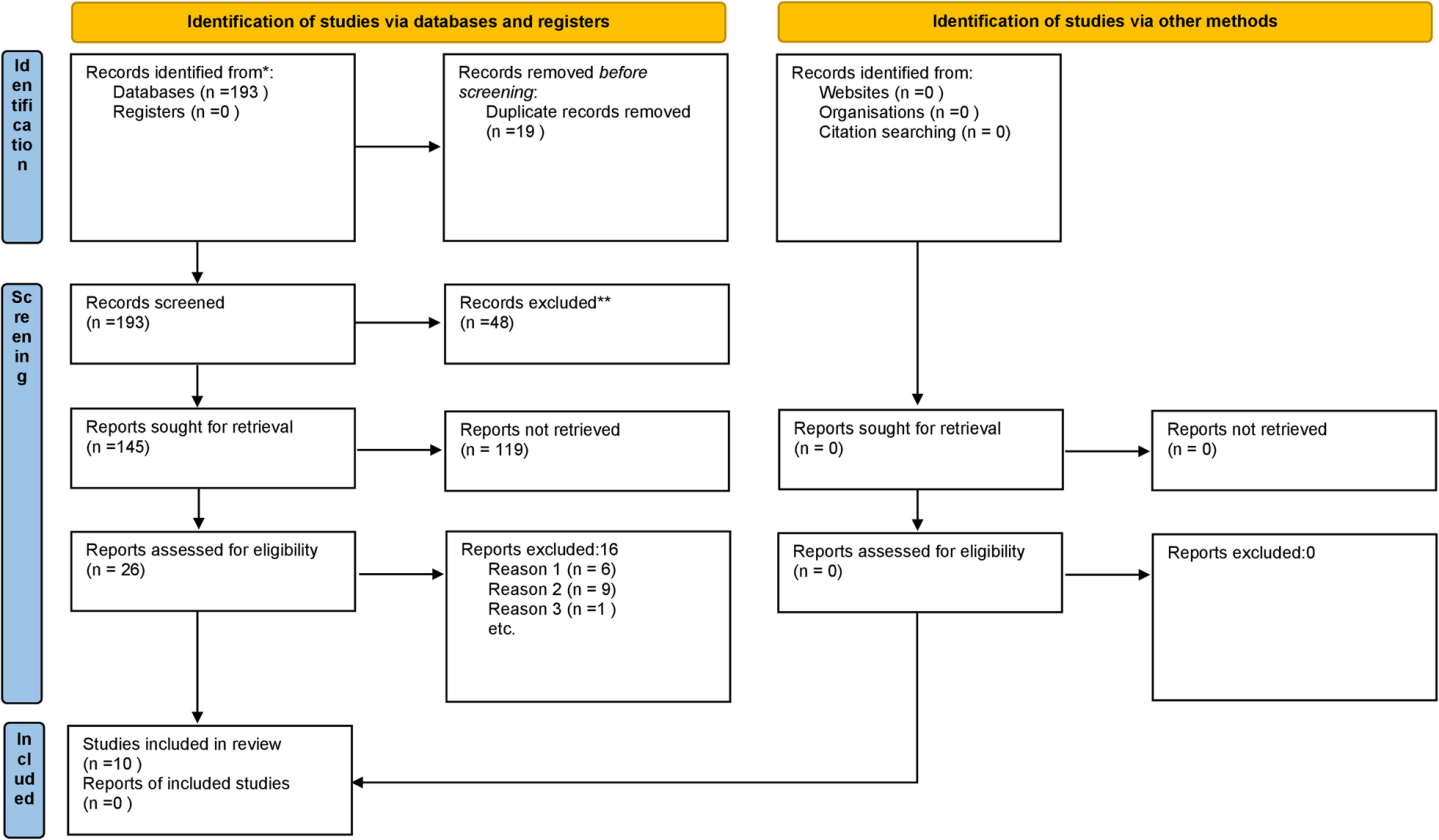
Study selection process

Figure 1 shows the four-phase study selection process. The initial search generated 193 records. After screening titles, abstracts and removal of duplicates, the full texts of 26 studies were reviewed. A total of ten studies were included in this review [17–26]. The reasons for the exclusion of the remaining 16 studies are presented in Additional file 2.

**Fig. 2:**
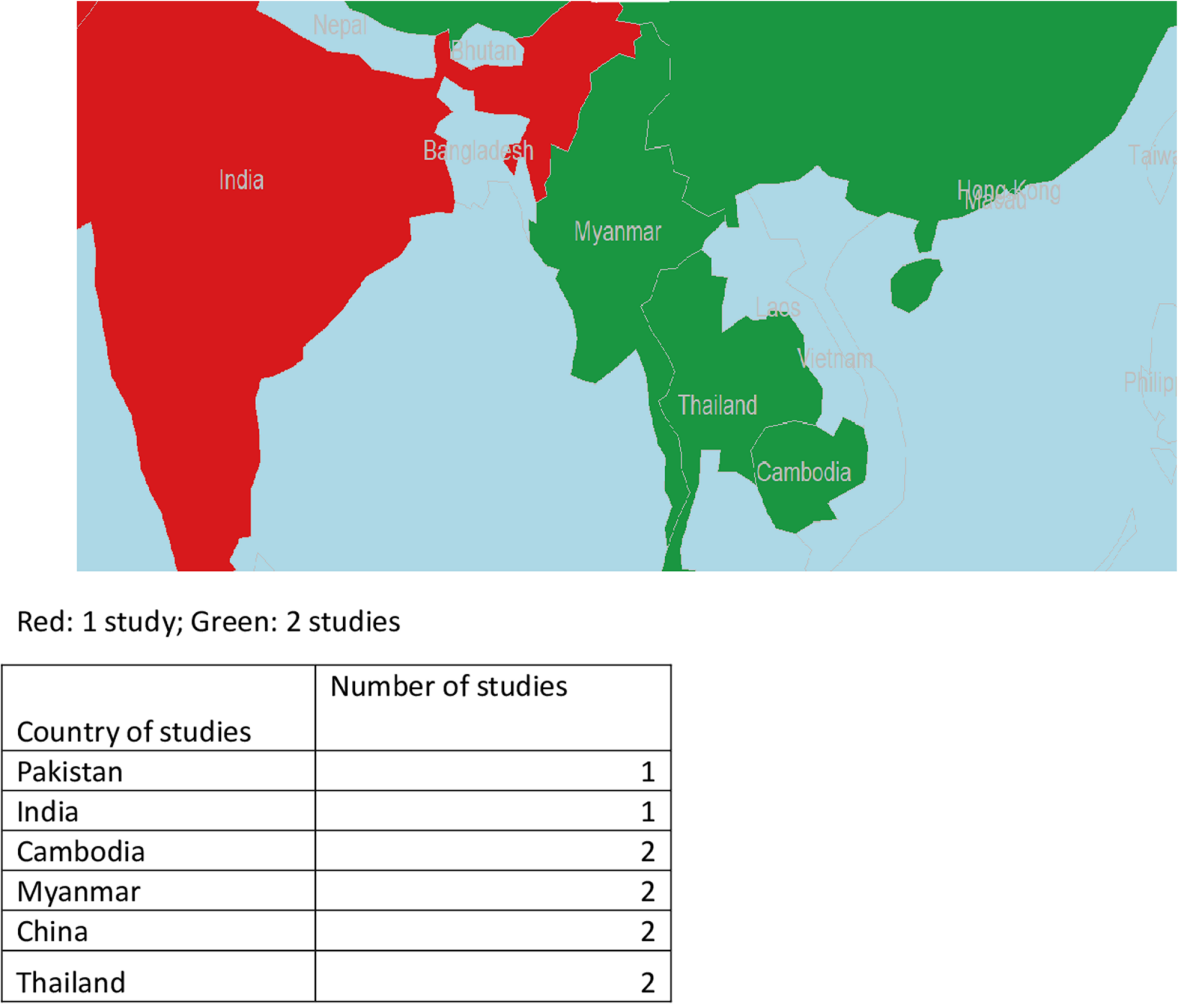
Geographic distribution of the included studies

The characteristics of the included studies are presented in Table 1. This review included 10 studies, comprising 10,945 adult participants with male predominance (56%). Subsets of all these 10 studies included the detection of asymptomatic cases in the surveillance context, using RDT in one study and microscopy in five studies; four studies were done using both tests [22, 24–26]. The diagnostic accuracies in all these studies were verified by PCR. Of these, one study was done under an RCD approach [23], while three studies were under an ACD approach [18, 19, 22] and six studies used a survey approach [17, 20, 21, 24–26]. The manufacturers of RDT used in the studies identified for the current analysis were different. Countries, where these studies were conducted were India [23], Pakistan [26], Thailand [18, 19], Cambodia [17, 21] and Myanmar [20, 25] and two studies in locations on either side of the China-Myanmar border [22, 24] (Fig. 2).

**Table 1:** Characteristics of included studies

Study, year	Study yr		Country	Approach	Age in yr	Male %	RDT/microscopy	PCR type	Remarks
Steenkeste, 2009 [17]		Sept 2001 +	Cambodia	CS	NA	NA	MC	Nested PCR	
Congpuong, 2012 [18]	2011,2012		Thailand	ACD	3–50	NA	MC	RT-PCR	
Rogawski, 2012 [19]	July 2011 +		Thailand	ACD	15–49 (84.7)	64.2	MC	RT-PCR	Based on index case in PCD
Wang, 2014 [20]	NA		Myanmar	CS	NA	NA	MC	Nested-PCR; RT-PCR	
Edwards, 2015 [21]	Aug 2013- March 2014		Cambodia	CS	15–40 (69.8%)	67.7	RDT(SD BIOLINE Malaria Ag P.f/P.v)	RT-PCR	
Li, 2016 [22]	May 2011—December 2012		China-Myanmar border	CS/ ACD/PCD	NA	NA	Myanmar border- MC Yunnan Province- RDT (Tycolpharm Co., Limited, UK)	Nested PCR	
van Eijk, 2016 [23]	2014		India	RCD	53	73	MC	cPCR	Age in median
Huang, 2017 [24]	June–Aug 2014		China-Myanmar border	CS	23.8 (0.5–80)	46.4	MC & RDT SD Malaria Ag P.f/P.v)	cPCR,RT-PCR,	Age in median & range
Zaw, 2017 [25]	2016		Myanmar	CS	32 (0.5–65)	54.4	MC & RDT (CareStart™ Malaria HRP2/pLDH)	RT-PCR	Age in median & range; study year is assumed from an ethical approval date
Naeem, 2018 [26]	Jan- Dec 2015		Pakistan	CS	NA	NA	MC & RDT(SD BIOLINE Malaria Ag P.f/Pan)	RT-PCR	

*ACD* active case detection, *cPCR* conventional PCR, *CS* cross-sectional study, *MC* microscopy, *PCD* Passive case detection, *RCD* Reactive case detection, *RDT* Rapid diagnostic test, *RT-PCR* Real time PCR, *ss-PCR* species-specific PCR, *yr* Year

### Quality of studies included

The methodological quality of each study included in this review is presented in Additional file 3. Most of the studies showed a moderate quality of bias, mainly due to inadequate information on blinding status in test examinations.

### Diagnostic accuracy

**Fig. 3:**
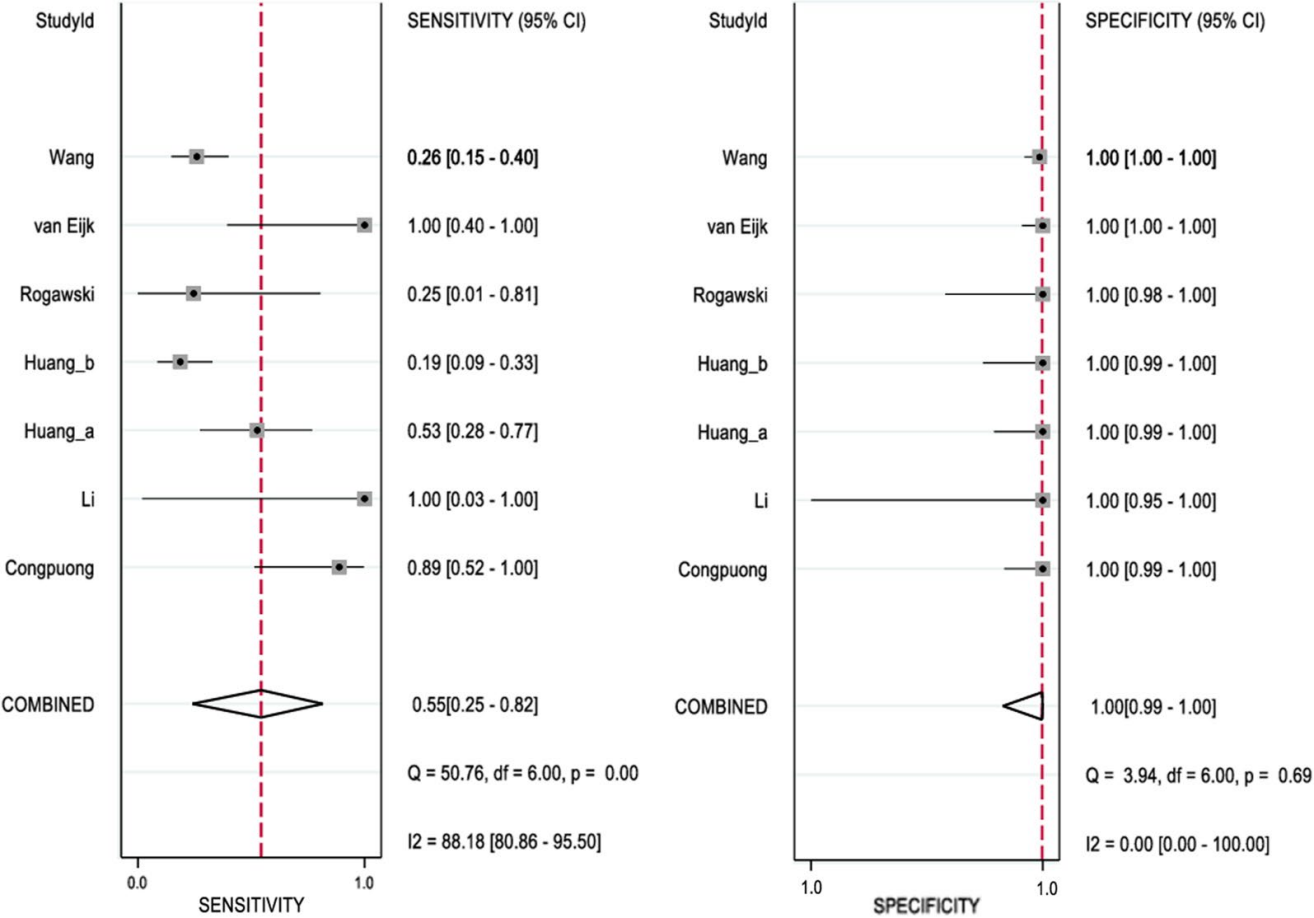
Forest plot of sensitivity and specificity of microscopy for *P. falciparum*

**Fig. 4:**
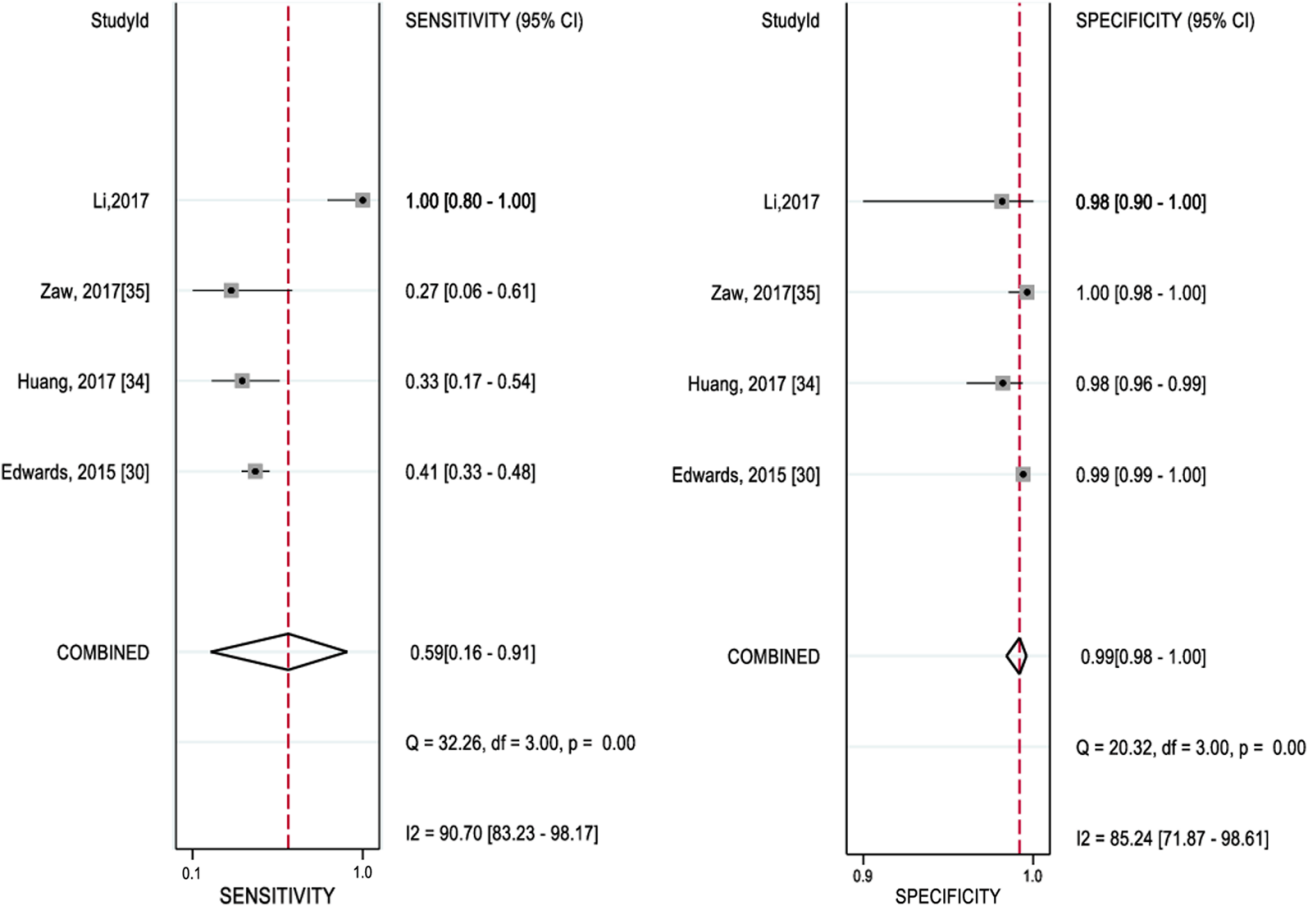
Forest plot of sensitivity and specificity of RDT for *P. falciparum*

Overall, there was a greater variation in sensitivity than specificity in the diagnostic tests evaluated. Sensitivity with the use of microscopy for detection of *P. falciparum* in asymptomatic participants varied widely, being from 19 to 100% and the summary estimate was 54% (95% CI: 11–92%). This was with substantial within-study heterogeneity (*I*^2^% 88.2%) (Fig. 3). There was also a wide variation in specificity from 27 to 100% with the use of RDT and the summary estimate was 59% (95%CI: 16–91%) (Fig. 4), This was also with low within-study heterogeneity (*I*^2^% 50.1%).

The use of microscopy for detection of *P. vivax* in asymptomatic participants varied from 9 to 100% and the summary sensitivity was 54% (95%CI: 11–92%). This was with substantial within-study heterogeneity (*I*^2^% 80%) (Additional File 4).

For RDTs that assessed *P. vivax* in asymptomatic participants, the pooled sensitivity was 51% (95%CI: 7–94%). There was substantial within-study heterogeneity (*I*^2^% 84%) (Additional File 5). These two diagnostic tests reported almost the same specificity, ranging from 98 to 100%.

Overall, between-test-comparison, for detection of *P. falciparum*, both tests were almost comparable. Within-test comparison also showed comparable accuracy*.* Although it was planned to perform subgroup analyses with RDT brand, study countries and PCR types, it was not possible due to the small number of studies.

## Discussion

The current study provides evidence on the accuracy of commonly and routinely used diagnostic tests for the detection of asymptomatic malaria cases in the surveillance context.

The major observations are as follows.

(i) This review consists of ten individual studies, in which blood samples were collected from 10,945 participants across six malaria-endemic countries in Asia.
(ii) The true positive rate of RDT, as well as microscopy, was relatively low in the detection of asymptomatic malaria cases, while the true negative rates were close to an ideal situation.

According to the Global Technical Strategy (GTS) 2016–2030 [2], 21 countries are aiming to eliminate malaria by 2020 and some countries by 2030. In the elimination context, all instances of detected parasitaemia (including gametocytaemia only) are considered a ‘malaria case’, regardless of the presence or absence of clinical symptoms [5]. The major concern is to detect sub-microscopic infection in the already low transmission areas. Earlier reviews on the use of microscopy [27] or RDTs [28, 29] reported a relatively higher diagnostic accuracy of microscopy than that of RDT for detection of *P. falciparum* in symptomatic malaria cases. This was not found in the present study that assessed asymptomatic malaria. This contradiction could be related to variations in parasite densities in symptomatic cases and asymptomatic cases. RDTs have a limit of detection around 200 parasites/μL [30]. It was assumed that other factors that may affect the accuracy of RDTs could include: deletion of target genes [31], methods of storage and handling of RDTs [30], varying concentrations of proteins in the blood, differences in manufacturing techniques [8] and objectivity/subjectivity in the interpretation of results were likely to be the same in the primary studies included in both reviews.

The present meta-analysis found that microscopy could miss 46% of the asymptomatic malaria infections in endemic areas for *P. vivax* and 45% for *P. falciparum* compared with PCR. A systematic review of sub-microscopic *P. vivax* infection reported that PCR detects 67% more *P. vivax* infections than microscopy in surveys of endemic populations [32]. The theoretical detection limit for standard thick film microscopy is approximately 4–10 parasites per ml, and for PCR it is 0.01–0.2 parasites per ml. In practice, a low number of parasitized red blood cells in a sample are often not sufficient to enable detection due to technical factors such as loss of parasites during staining of the microscopy slides [5]. Microscopy is likely to miss lower-density infections during the screening of endemic populations [27]. Moreover, studies have highlighted that observed parasite densities can decrease with age, immunity, and on a technique basis, by loss of parasites during staining [33]. Additionally, microscopists’ competency can deteriorate when they are not regularly examining a reasonable number of slides [7]. Increasing the specificity of the diagnostic method used in the field will lead to significantly reduced numbers of individuals being treated but would not reduce the proportion of the reservoir identified [34]. The higher accuracy of RDT in detecting *P. falciparum* compared with microscopy in the present meta-analysis study was likely to be due to persistent antigenaemia, rather than to the presence of current infections among these participants.

Concerning the methodology, the advantage of the *I*^2^ test is that it does not inherently depend on the number of studies in the meta-analysis [15]. The substantial *I*^2^ values for both RDT and microscopy as shown in this study indicated the variability of diagnostic accuracy among different populations. As mentioned above, factors such as sample type (volume of blood samples), age of patients and immune status of the individuals/community were likely to impact the heterogeneity of the results. Due to a paucity of data, we were unable to quantify the impact of these factors.

## Implications

Targeted screening for asymptomatic infections may have a potential role in accelerating elimination and reducing the probability of reintroduction and resurgent transmission in these populations [37]. Across all levels of transmission intensity, a substantial proportion of malaria infections are asymptomatic and often present at pathogen densities below the threshold for detection by microscopy or RDTs [8, 38, 39]. The currently and routinely used diagnostic tools are limited in their performance for asymptomatic malaria, as shown in this analysis. The urgency and importance of quickly obtaining results from the examination of peripheral blood samples from asymptomatic malaria cases need more sensitive methods for malaria diagnosis, although those that are presently available are impractical for routine laboratory use. For example, molecular diagnostic methods such as PCR have improved the detection of asymptomatic parasitaemias, but the technique requires demanding thermocycling conditions and that is not feasible for the hand-held pathogen detection in a field setting [38] nor at the point-of-care settings. At the last stage of malaria elimination, programmes must be highly vigilant and seek to find every infection [40, 41]. In this context, policymakers may be interested in the comparative accuracy of available diagnostic tests as well as in additional information about the costs and logistics of testing to assist in evidence-based decisions regarding diagnosis and treatment policies and procurement guidelines.

Due to the limited data available in the studies included, and the scope of this review, it is not possible to provide that additional information from the current analysis. But it is to be noted that FN- RDT results have severe consequences in malaria-endemic settings because negative RDT results are not routinely confirmed by any other diagnostic tests [42]. FP results from a specific population with fever due to other infectious agents (e.g., leishmania, dengue virus, Chagas trypanosomes, or schistosome) in endemic areas is also a concern [43]. The prevalence of symptomatic patients carrying pfhrp2-deleted parasites (causing FN HRP2 RDT results) is ≥ 5% [44], albeit with spatial heterogeneity. A study reported a greater increase in the risk of an FN-RDT result as prevalence decreases [45], which might be an example for asymptomatic malaria with a low parasite burden. Hence, it is also important to rule out any suspected quality defect with the malaria RDTs or low parasite densities as a cause of the FN. The spatial variation in FN results provides an insight into health care provision regarding the usefulness of malaria RDTs in different settings and their implication for the treatment of suspected malaria cases. As there was high specificity in the present study, the issue of FN-RDT was assumed not to be a major challenge in the study areas. A study reported that the FN results for *P. falciparum* specimens occurred at low, medium and high parasite densities proving that parasitaemia did not influence the false negativity [46].

Ultrasensitive RDTs (uRDTs) have been proposed as a tool to detect malaria infections even at low-density infections (below 100–200 parasites/µL) through mass test and treat (MTAT) campaigns, or proactive or reactive testing and treating [47]. However, studies reported that uRDTs did not provide any further benefits in the detection of infections among febrile individuals in the community when compared to regular RDTs [47, 48].

In malaria hotspots, it is important to be able to identify and treat these infections [21]. A combination of PCR-based strategies can be practical and effective active surveillance tools for asymptomatic malaria in countries of endemicity [20]. Overall, targeted “customised” interventions and surveillance activities should be implemented in these sites to accelerate elimination efforts in the region [21].

## Study limitations

There are several limitations to the present study. The current results are limited by the quality of primary diagnostic test accuracy studies. As most individual studies included in this review did not report on whether they had performed a blinded index/reference test interpretation, over/underestimation of true accuracy is a concern. With regard to the reference test PCR, a common concern is the potential influence of contamination in these low-transmission areas as the contamination rates of 0.7%–10% has been reported for the laboratories encountered [35]. Moreover, malaria DNA released from dead parasites only marginally affects the validity of the positive results [36]. Studies are needed to provide more details of “asymptomatic", as these cases may often only be asymptomatic at the day of data collection and have had or will have mild symptoms. Furthermore, there are several forms of PCR used for the diagnosis of *Plasmodium* infections. The substrate can come from dried blood spots (DBS), venous blood samples, or in the case of high-volume PCR a cryoprecipitate The lower limit of detection varies by orders of magnitude. This information was not described in many of the studies included. Hence, it is not possible to stratify by a different form of PCR used.

As *Plasmodium malariae* is increasing in the proportion of all malaria and *Plasmodium ovale* is also identified in some areas, additional tests that detect *P. malariae* and/or *P. ovale* are needed as a priority in the malaria elimination context. The increasing role of *Plasmodium knowlesi* as a contributor towards fever due to zoonotic malaria may require the availability of field-ready diagnostics specific to this species.

## Conclusion

The findings of this meta-analysis suggest that RDTs and microscopy have limited sensitivity and are inappropriate for the detection of asymptomatic *Plasmodium* infections. Other methods including a combination of PCR-based strategies, Loop-Mediated Isothermal Amplification (LAMP) technique must be considered to target these infections, in order to achieve malaria elimination. However, more data is needed for the wide acceptance and feasibility of these approaches. Studies to explore the role of asymptomatic and sub-patent infections in the transmission of malaria are of critical importance and are recommended.

## Supplementary Information

**Additional file 1: Table S1**. PRISMA-DTA Checklist.

**Additional file 2: Table S2**. Summary of excluded studies.

**Additional file 3: Figure S1**. Summary of the methodological quality assessment across all studies

**Additional file 4: Figure S2**. Forest plot of sensitivity and specificity of microscopy for* P. vivax*

**Additional file 5: Figure S3**. Forest plot of sensitivity and specificity of RDT for* P. vivax*

## Data Availability

All data generated or analysed during this study are included in this article and its supplementary information files.

## Abbreviations

AUC: Area under the curve
CI: Confidence interval (CI)
GTS: Global Technical Strategy
OR: Odds ratio
*P. falciparum*: *Plasmodium falciparum*
*P. vivax*: *Plasmodium vivax*
RDT: Rapid-onsite diagnostic test
