# Supplementary material for: Detection of asymptomatic malaria in Asian countries: a meta-analysis of diagnostic accuracy
Source: Malar J. 2022 Feb 16;21:50. doi: 10.1186/s12936-022-04082-0 (PMC8848787; doi:10.1186/s12936-022-04082-0)
Supplement: Supplementary file 1 — Additional file 1: Table S1. PRISMA-DTA Checklist. [file 12936_2022_4082_MOESM1_ESM.doc]

Additional File 2: Table S1 Summary of excluded studies

| No. | Study | Citation. | Main reason |
| --- | --- | --- | --- |
| 1 | Atkinson, 2012 | Atkinson J-A, Johnson M-L, Wijesinghe R, et al. 2012. Operational research to inform a sub-national surveillance intervention for malaria elimination in Solomon Islands. Malar J 2012, 11:101 | Not in Asia, mainly with symptomatic cases |
| 2 | Kattenberg,2012 | Kattenberg,JH, Tahita CM, Versteeg IAJ, et al. Evaluation of antigen detection tests, microscopy, and polymerase chain reaction for diagnosis of malaria in peripheral blood in asymptomatic pregnant women in Nanoro, Burkina Faso. Am J Trop Med Hyg 2012; 87: 251-256. | Not in Asia, Pregnant women |
| 3 | Nyunt,2013 | Nyunt MH, Kyaw MP, Win KK,et al.. Field evaluation of HRP2 and pan pLDH-based immunochromatographic assay in therapeutic monitoring of uncomplicated falciparum malaria in Myanmar. Malar J. 2013;12:123. | Not asymptomatic cases |
| 4 | Hsiang, 2012 | Hsiang MS, Hwang J, Kunene S, et al. Surveillance for malaria elimination in Swaziland:a national cross-sectional study using pooled PCR and serology. PLOS One 2012; 7: e29550. | Difficult to extract 2x2 accuracy data |
| 5 | Hsiang, 2014 | Hsiang MS, Greenhouse B, Rosenthal PJ. Point of care testing for malaria using lamp, loop mediated isothermal amplification. J Infect Dis 2014;210: 1167 | No data on diagnostic accuracy |
| 6 | Stresman, 2015 | Stresman GH, Baidjoe AY, Stevenson J, et al. Focal Screening to identify the subpatent parasite reservoir in an area of low and heterogeneous transmission in the Kenya highlands. J Infect Dis 2015;212:1768–1777. | Not in Asia, No data on diagnostic accuracy |
| 7 | Laban, 2015 | Laban NM, Kobayashi T, Hamapumbu H, et al. Comparison of a PfHRP2-based rapid diagnostic test and PCR for malaria in a low prevalence setting in rural southern Zambia: implications for elimination. Malar J. 2015;14:25. | Symptomatic cases were included |
| 8 | Yukich, 2017 | Yukich J, Bennett A, Yukich R, et al. Estimation of malaria parasite reservoir coverage using reactive case detection and active community fever screening from census data with rapid diagnostic tests in southern Zambia: a re-sampling approach. Malar J, 2017; 16, 317. 50. | Not in Asia |
| 9 | Wang,2017 | Wang D, Cotter C, Sun X, et al. Adapting the local response for malaria elimination through evaluation of the 1-3-7 system performance in the China–Myanmar border region. Malar J, 2017; 16, 54. | No data on diagnostic accuracy |
| 10 | Nyunt,2018 | Nyunt, MH, Soe TN, Shein T, et al. Estimation on local transmission of malaria by serological approach under low transmission setting in Myanmar. Malar J, 2018; 17, 6. | No data on diagnostic accuracy |
