# Supplementary material for: Detection of asymptomatic malaria in Asian countries: a meta-analysis of diagnostic accuracy
Source: Malar J. 2022 Feb 16;21:50. doi: 10.1186/s12936-022-04082-0 (PMC8848787; doi:10.1186/s12936-022-04082-0)

Additional File 3: Figure S1: Summary of the methodological quality assessment across all studies


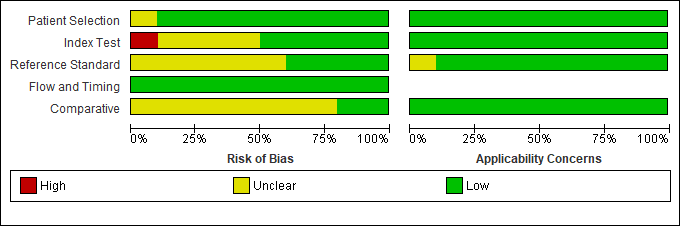


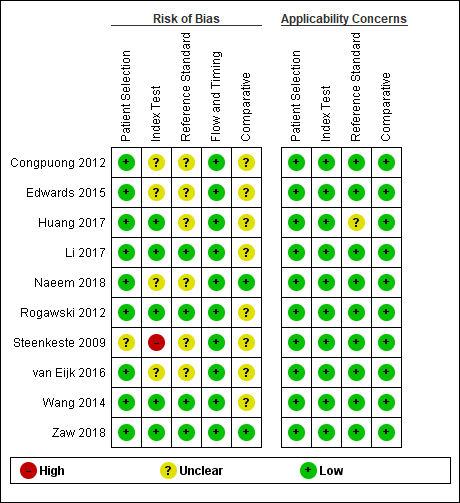

Supplement: Supplementary file 3 — Additional file 3: Figure S1. Summary of the methodological quality assessment across all studies [file 12936_2022_4082_MOESM3_ESM.doc]
