# Supplementary material for: Detection of asymptomatic malaria in Asian countries: a meta-analysis of diagnostic accuracy
Source: Malar J. 2022 Feb 16;21:50. doi: 10.1186/s12936-022-04082-0 (PMC8848787; doi:10.1186/s12936-022-04082-0)
Supplement: Supplementary file 4 — Additional file 4: Figure S2. Forest plot of sensitivity and specificity of microscopy for P. vivax [file 12936_2022_4082_MOESM4_ESM.doc]

Additional File 4: Figure S2 Forest plot of sensitivity and specificity of microscopy for *P. vivax*
